# 

**Published:** 1877-01

**Authors:** 


### CONTENTS.

PAGE.

**CONTRIBUTIONS.**

A Doctor's Responsibility. BEN. H. PRATT............197
Nerve-Storms. P. H. HAYES, M. D........................199

**MEDICAL ITEMS AND NEWS.**

Antidote to Strychnia—Toothache—Hemorrhage—Obesity—-Neuralgia (2)—Asthma (2)—Uterine Catarrh—Coca—-Indication of Death—-Dropsies—Pigmentary Stains—-Enlargement of the Spleen—Febrifuge—-Chronic Intermittents—-Linimentum Chloriformi—Buck's Burn Mixture—Lotion for Urticaria—-Aortic Aneurism—-Salicylate of Ammonium—-Concentrated Solution of Salicylic Acid—Relief of Cancer—Anti-Salivation Powder—-Strumous and Syphilitic Ulcers—Emmenagogue Pill—Enlarged Spleen—Sedative Solution—Pruritis—-Preservation of Syrups—-Acute Rheumatism—Chloral Cream—-Syphilitic Buboes—Chloral Plaster—-Poisoning by Toxicodendron—-Instantaneous Synapisms—Embalming—Sore Nipples-—Diabetic Subjects—-Polyuria-—Intestinal Obstructions—Burns—Whooping Cough—-Rickets—Epilepsy-—Cystitis—Gonorrhœa—Puerperal Convulsions—-Glandular Swellings—Erysipelas (3)—-Selected Hospital Formula—-Sea Sickness—-Primary Diseases of the Heart—Relief of Pain—Chronic Gastro-Intestinal Catarrh—Ergot of Rye—-Solution of Glycerine and Water—Necrosis—Diabetes Insipidus—Diarrhœa..........202 to 214

PAGE

**OUR CORRESPONDENCE.**

Four Letters with Replies ........................215

**HOUSEHOLD HINTS AND RECIPES.**

Cement for Vessels—-Inkstains—-Fasten Iron to Wood—Restore Color—-Progress of Hippophagy—Marking Ink—Mucilage for Labels—Perspiration of Feet—-Adhesive Plaster—Infant Food—-Copying Pencils—-Closing Cracks in Stoves—-Preparation for Washing Blue—Toothpowder—-English Court Plaster—Elastic Dammar Varnish—Poisonous Cooking Utensils........................216-217

**EDITORIAL CHIT-CHAT.**

Pay Up—Fine Singing—Street Cars—A Vacancy—W. R. Warner & Co.—Hideous Chromos—Flora Culture—Johnson—Personal—The Difference—Micro-Photographs—Time—Charity Patients—Presidential Muddle—The Zoo—A Balancing Effort—Dr. Eldridge—-Sore Finger—-A Perplexed Countryman—Christmas—The Doctor's Conservatory..............................221,324

